# RETRACTION: Ameliorative Effects of Scopoletin from *Crossostephium chinensis* against Inflammation Pain and Its Mechanisms in Mice

**DOI:** 10.1155/ecam/9782815

**Published:** 2026-02-27

**Authors:** Evidence-Based Complementary and Alternative Medicine

RETRACTION: T.‐N. Chang, J.‐S. Deng, Y.‐C. Chang, et al., “Ameliorative Effects of Scopoletin from *Crossostephium chinensis* against Inflammation Pain and Its Mechanisms in Mice,” *Evidence-Based Complementary and Alternative Medicine*, vol. 2012 (2012), https://doi.org/10.1155/2012/595603.

The above article, published online on 6 September 2012 in Wiley Online Library (https://wileyonlinelibrary.com), has been retracted by John Wiley & Sons Ltd.

The retraction has been agreed following an investigation of the concerns raised by *Actinopolyspora biskrensis* on PubPeer [1], which identified several concerns related to Figure 4a. More specifically, the control panel overlaps with the control panels of Figure 6a of [2] and Figure 8a of [3]. Additional similarities have been identified between the Carr panel and the similarly labelled panels in Figure 8a of [4] and Figure 4a of [5].

The experimental conditions for the papers differ in the number of animals used and methods of image acquisition.

As a result of the investigation, the data and conclusions of this article are considered unreliable.

The authors have been informed of this decision but did not provide a response.
